# Neurofilament light is a novel biomarker for mitochondrial encephalomyopathy, lactic acidosis, and stroke-like episodes

**DOI:** 10.1038/s41598-021-81721-7

**Published:** 2021-01-21

**Authors:** Yong-Sheng Zheng, Chong Sun, Rong Wang, Ne Chen, Su-Shan Luo, Jian-Ying Xi, Jia-Hong Lu, Chong-Bo Zhao, Yu-Xin Li, Lei Zhou, Jie Lin

**Affiliations:** 1 Department of Neurology, Huashan Hospital, Fudan University, 12 Middle Urumqi Road, Shanghai, 200040 China; 2 Department of Radiology, Huashan Hospital, Fudan University, 12 Middle Urumqi Road, Shanghai, 200040 China

**Keywords:** Genetics, Molecular biology, Neuroscience, Biomarkers, Diseases, Medical research, Neurology

## Abstract

Mitochondrial encephalomyopathy, lactic acidosis, and stroke-like episodes (MELAS) is a complicated maternally inherited disorder lacking of sensitive and specific biomarkers. The objective of this study was to investigate the serum neurofilament light chain (NfL) as a novel biomarker of neurological dysfunction in MELAS. Patients with different status of MELAS were enrolled in this study. The Mini-Mental State Examination (MMSE) was given to the participants to evaluate cognition status. Multiple functional MRI was performed on the participants. Blood samples were collected and the serum NfL concentrations were determined by the single-molecule array technology (Simoa). This study enrolled 23 patients with MELAS, 15 people in the acute attack phase of MELAS and 10 people in the remission phase, including 2 patients in both acute attack and remission phase. Sixteen healthy controls (HCs) were also enrolled. Serum NfL level increased significantly in patients with MELAS. Serum NfL level in the acute attack group (146.73 [120.91–411.31] pg/ml, median [IQR]) was higher than in the remission group (40.31 [19.54–151.05] pg/ml, median [IQR]) and HCs group (7.70 [6.13–9.78] pg/ml, median [IQR]) (p < 0.05). The level of NfL in the remission phase group was higher than in HCs group (p < 0.05). A negative correlation was found between the serum NfL level and MMSE (p = 0.006, r = -0.650). The NfL concentration correlated positively with stroke-like lesion volume in the brain (r = 0.740, p < 0.001). Serum NfL may serve as a novel biomarker for the neurological dysfunction in MELAS patients.

## Introduction

Mitochondrial encephalomyopathy, lactic acidosis, and stroke-like episodes (MELAS) is a complicated maternally inherited disorder first described in 1984^1^. For a long time, effective biomarker of MELAS was rare for diagnosis and evaluation of disease status, including in acute episodes and remission. Previously, fibroblast growth factor 21, growth differentiation factor 15, lactate acid, and brain imaging were used as biomarker of MELAS^2–5^.

A biomarker is still needed with several criteria including sample accessibility, a strong correlation between the biomarker and brain MRI that reflects the functional deficiency of the brain and that it is able to differentiate between MELAS stages.

Making up the cytoskeleton of axons, neurofilament light (NfL) is one of the most important structure proteins in neurons^6^. It has been reported as a promising biomarker in varieties of neurological disorders^7–9^. Indeed, serum NfL concentration was strongly correlated with the NfL level in CSF^10^. Serum NfL as a non-invasive biomarker reflected the expression of NfL in CSF for neurological disorders^11–13^. An encouraging result has shown that the NfL level in CSF was significantly elevated in patients with mitochondrial diseases^14^. However, serum NfL levels have never been explored specially in MELAS. In this article we explored NfL as an effective biomarker with the following properties: (1) serum NfL level elevated in patients with MELAS and; (2) serum NfL may be correlated with the neurological dysfunction in patients with MELAS.

## Results

### Clinical characteristics

This cross-sectional study enrolled 23 patients with MELAS, 15 people in the acute attack phase and 10 people in the remission phase, as well as 16 HCs. The demographic data and clinical information for all patients was summarized on Table 1. The m.3243A>G mutation in the *MT-TL1* gene was found in 19 patients, but one had a m.1621A>G mutation. Another 3 patients with negative results in mitochondrion gene test were diagnosed with MELAS by muscle biopsy due to the presence of ragged-red fiber by modified Gomori's trichrome stain and Succinic dehydrogenase (SDH) staining. No significant differences were found by age and gender (Table 2).

**Table 1:** Clinical characteristics of patients with MELAS.

Number	Age	Sex	Mitochondrial gene	Disease status	NfL concentration (pg/ml)	Lesions location	Lesions volume (mm^3^)	MMSE
1	25	M	m.3243A>G	A	146.74	Bilateral temporal lobes	72,252.99	28
2	45	M	m.3243A>G	A	411.31	Bilateral occipital lobes	58,670.72	3
3	20	M	m.1621A>G	A	901.07	Right occipital lobe	NA^a^	ND
4	45	F	m.3243A>G	A	124.80	ND	NA^b^	ND
5	32	M	m.3243A>G	A	219.76	Left parietal lobe	102,230.36	ND
6	49	F	N	A	140.20	ND	NA^b^	ND
7	23	M	m.3243A>G	A	35.57	Right occipital lobe	28,832.51	30
8	16	F	m.3243A>G	A	287.09	Right parietal and occipital lobes	41,349.06	24
9	26	M	m.3243A>G	A	128.60	Left parietal and occipital lobes	95,702.81	18
10	52	M	N	A	1031.42	Right parietal, occipital and temporal lobes	NA^a^	ND
11	61	M	N	A	120.91	Left temporal lobe	NA^a^	16
12	10	M	m.3243A>G	A	81.19	Left temporal lobe	1506.78	30
13	27	M	m.3243A>G	A	180.90	Left parietal, occipital, temporal and right temporal lobes	128,424.60	9
14	51	F	m.3243A>G	A	470.96	Right temporal lobe	84,462.97	ND
15	25	M	m.3243A>G	A	21.07	No lesion	0.00	ND
16	22	M	m.3243A>G	R	36.60	Left periventricular	14,373.78	27
17	20	M	m.3243A>G	R	32.50	Bilateral temporal lobes	19,453.45	27
18	27	M	m.3243A>G	R	166.61	ND	NA^b^	26
19	16	F	m.3243A>G	R	153.64	Left temporal and right occipital lobes	30,407.81	28
20	29	M	m.3243A>G	R	44.03	Bilateral temporal lobes	21,044.23	ND
21	50	F	m.3243A>G	R	120.41	Left parietal, occipital and temporal lobes	12,945.54	19
22	42	M	m.3243A>G	R	16.17	ND	NA^b^	ND
23	51	F	m.3243A>G	R	20.67	No lesion	0.00	23
24	25	F	m.3243A>G	R	150.19	Bilateral temporal lobes	14,003.95	10
25	23	M	m.3243A>G	R	3.53	No lesion	0.00	30

*MELAS* mitochondrial encephalomyopathy, lactic acidosis, and stroke-like episodes, *NfL* neurofilament light, *lesions volume* stroke-like lesions volume, *M *male, *F* female, *A* patients with MELAS in the acute attack phase, *R* patients with MELAS in the remission phase, *ND* not done, *N* negative, *NA* not available, *MMSE* Mini-Mental State Examination, *a* not performing multi-functional MRI, *b* not experiencing MRI.

**Table 2:** Demographics for the study population.

Variable	HCs	A	R	p value
n	16	15	10	
Age, mean (SD)	40.8(13.9)	33.8(14.8)	30.5(11.9)	0.136
Male, %	56.3%	73.3%	60%	0.270
Serum NfL median (IQR) pg/ml	7.70 (6.13–9.78)	146.73 (120.91–411.31)	40.31 (19.54–151.05)	< 0.001

*HCs* healthy controls, *A* patients with MELAS in the acute attack, *R* patients with MELAS in the remission phase, *NfL* neurofilament light.

### Serum NfL feature in patients with MELAS and healthy controls

**Figure 1:**
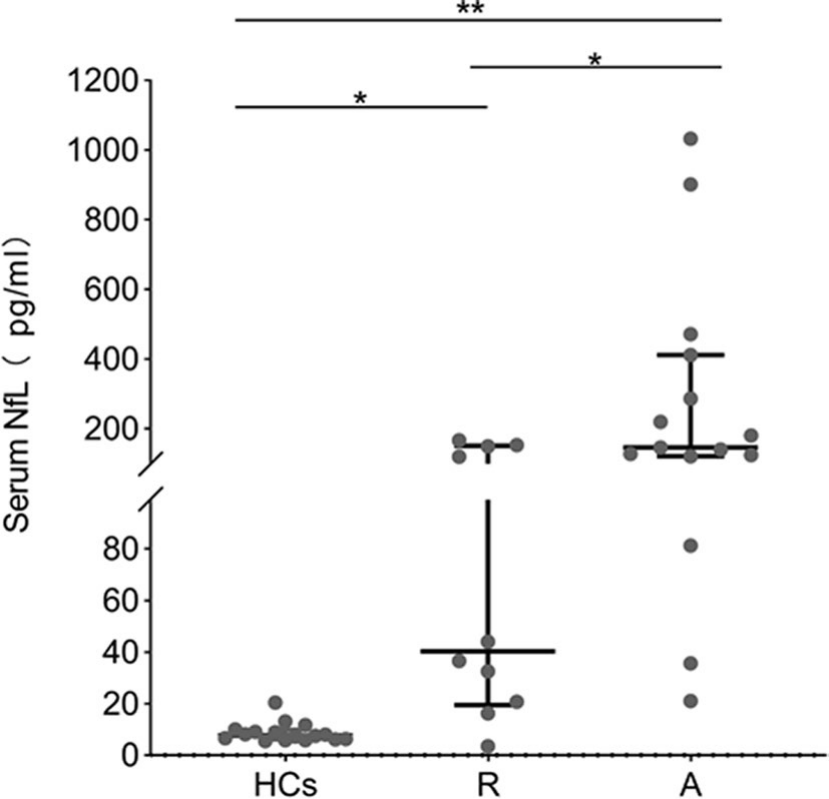
Serum NfL levels in all study participants. *NfL* neurofilament light, *HCs* healthy controls, *A* patients with MELAS in the acute attack, *R* patients with MELAS in the remission phase.

Serum NfL was measured in 23 patients with MELAS (Table 1) and 16 HCs. Serum NfL levels increased significantly in patients with MELAS. The average of the NfL levels was higher in the acute attack group (146.73 [120.91–411.31] pg/ml, median [IQR]) than in the remission phase (40.31 [19.54–151.05] pg/ml, median [IQR]) and HCs (7.70 [6.13–9.78] pg/ml, median [IQR]) (p < 0.05, Fig. 1). Serum NfL concentrations in the patients with MELAS in the remission phase were higher than the HCs group (p < 0.05, Fig. 1). No significant differences were found between NfL concentrations and age and gender.

### Correlation between cognitive evaluation and serum NfL

**Figure 2:**
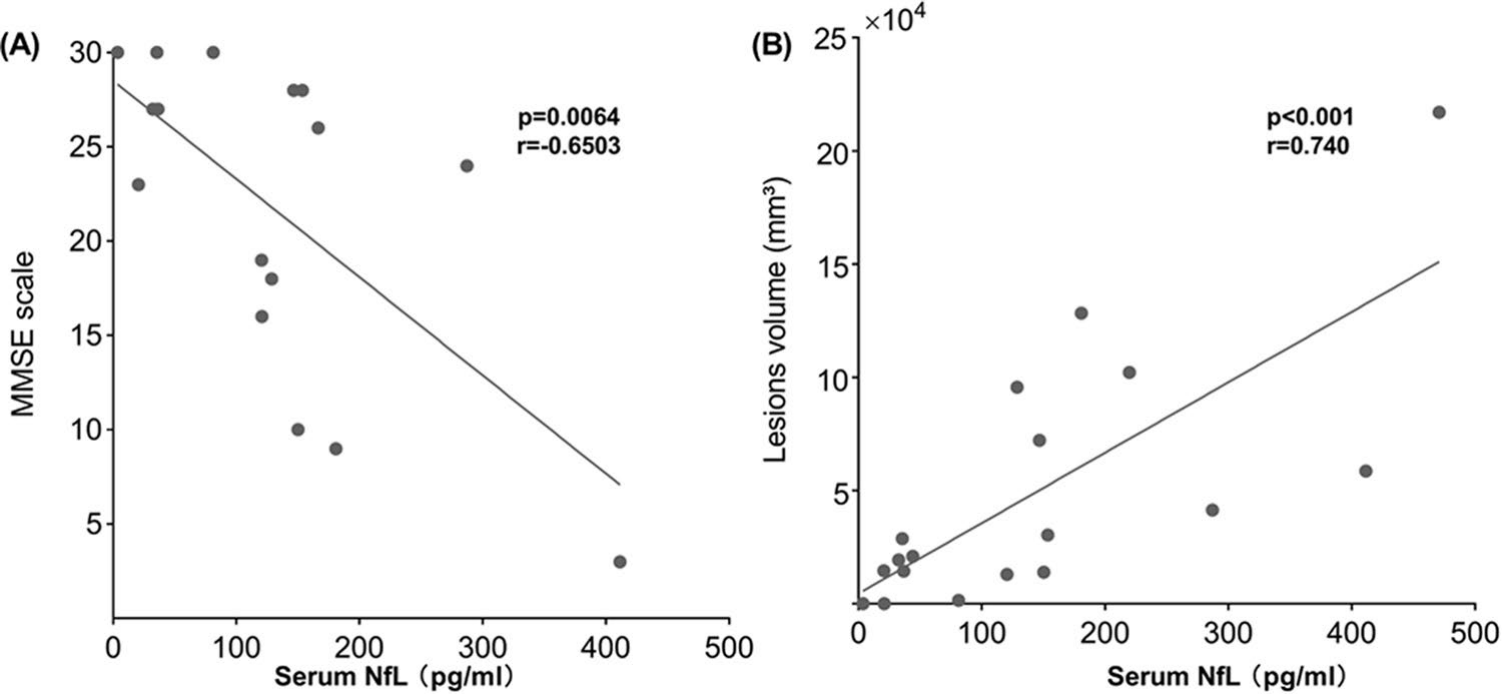
Correlations of serum NfL level with MMSE and stroke-like lesion volume. (**A**) The association of between MMSE scale and serum NfL level; (**B**) the relation between serum NfL level and stroke-like lesion volume. *MMSE* Mini-Mental State Examination, *NfL* neurofilament light, *lesion* *volumes* stroke-like lesion volumes.

Because of cortical injuries or severe neurological dysfunction in the acute attack group, a number of patients were not able to cooperate with the assessment procedures. Only 16 affected individuals performed the assessment test. However, a significant correlation was found between the serum NfL level and cognitive dysfunction (p = 0.006, r = − 0.650; Fig. 2A).

### NfL level fluctuation and lesions volume change on MR imaging

All patients included in this study had documented stroke-like episodes. MR scanning were performed on a total of 18 patients. Three patients in the acute attack performed conventional MRI without multi-functional MRI and 2 patients in the acute attack did not perform MR scanning but had newly acute neurological deficits. Two patients in the remission did not perform MR scanning during follow up. The SLLs volumes in patients who experienced both conventional and multi-functional MRI were calculated and were shown on Table 1. The serum NfL level positively correlated with SLLs volume (r = 0.740, p < 0.001; Fig. 2B).

### The trends of NfL level and MR imaging in two follow-up patients with MELAS

**Figure 3:**
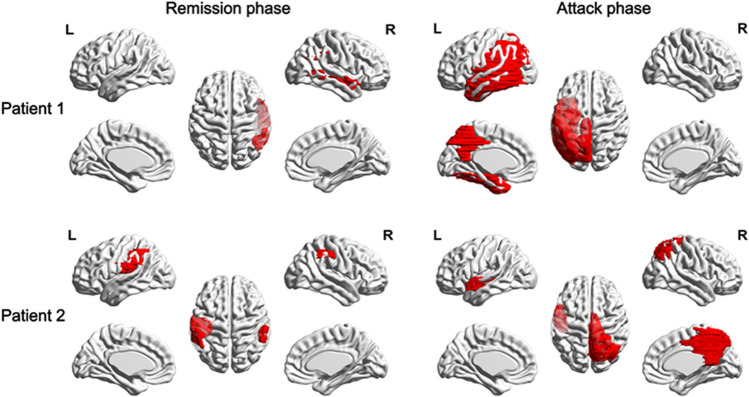
Stroke-like lesion in two follow-up patients. The red parts of brain shown in picture are the SLLs areas; *NfL* neurofilament light, *lesion volume* stroke-like lesion volume, *L* left hemisphere, *R* right hemisphere.

**Figure 4:**
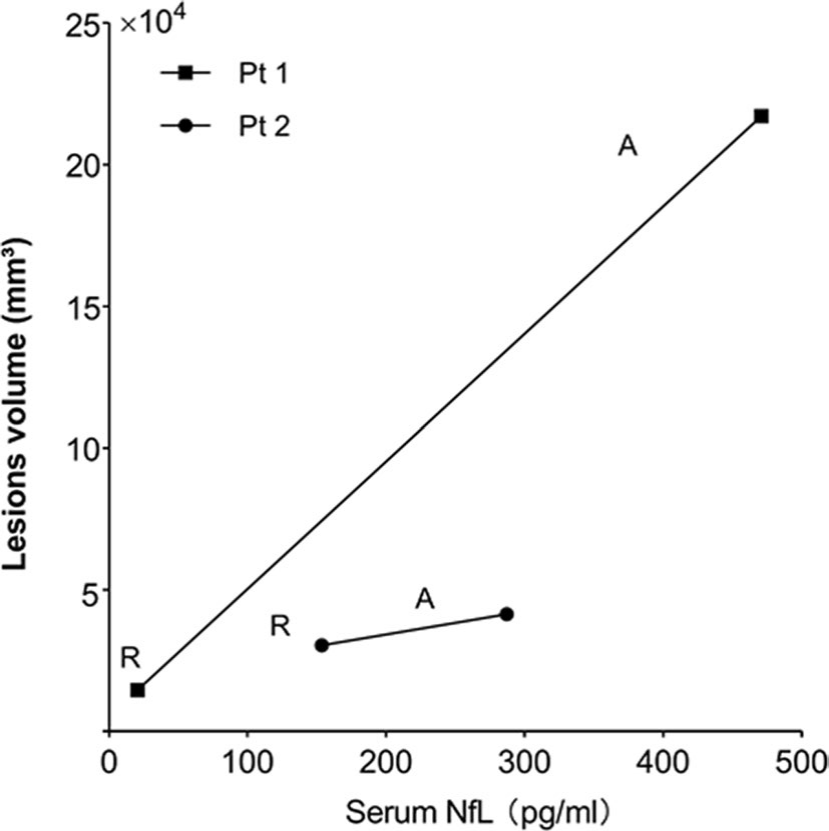
NfL concentration and Stroke-like lesion volume from remission phase to acute attack in two follow-up patients. *NfL* neurofilament light, *A* patients with MELAS in the acute attack, *R* patients with MELAS in the remission phase, *Pt 1* patient 1, *Pt 2* patient 2.

The SLLs of two patients, both of whom had experienced attack and remission phases, were shown in the Fig. 3. In patient 1, small sections of the lesions were found on the right temporal lobe in the remission phase. However, massive SLLs were observed on left temporoparietal lobe and occipital and frontal lobe in attack phase. In patient 2, there were a few left lesions after the previous attack on the left and right temporal lobes. However, larger area SLLs arose on the left superior temporal gyrus and right parietal-occipital areas in the acute attack episode. The lesions volume of two patients in attack phase were predominantly higher than in the remission phase. In contrast, from the remission to attack phase, serum NfL level linearly increased markedly, which was correlated with the lesions volume (Fig. 4).

## Discussion

The serum NfL levels differed in patients with MELAS. Higher serum NfL levels were associated with severe cognition impairment measured in the MMSE section in this study. Importantly, serum NfL levels were correlated with stroke-like lesions volume in a positive manner. Together, these findings highlight that serum NfL may be a sensitive biomarker of neurological dysfunction in MELAS.

NfL has been proposed as a biomarker for axonal injury in a number of neurological disorders^9, 11, 15–17^. NfL as a tool for assessing the functional consequences in mitochondrial diseases has been rarely mentioned in the peer-reviewed literature. Noteworthy, elevated NfL levels in CSF samples from patients with mitochondrial diseases was recently reported. In that study, ELISA technology for NfL detection was less sensitive and the lumbar puncture sample collection was invasive^14^. Compared to ELISA, Simoa is 126-folds more sensitive^18^. Thus, we employed Simoa in our study for its sensitivity and specificity. Serum NfL levels were found to be higher in patients with MELAS both in the acute attack and in remission phases compared to HCs. This mainly resulted from the accumulation of acute injuries in cortical areas and cortical white matter where NfL primarily expressed. Meanwhile, serum NfL levels were higher in the acute attack group than in remission group. This is consistent with clinical practice where a more severe cognition impairment and worse disability were observed in the acute attack group of patients with MELAS compared to those in remission. However, the correlation between the NfL levels and cognition status still needs to be elucidated.

Research was reported in patients with AD that high plasma NfL level was correlated with worse score in MMSE^11^. This implied that MMSE could be used for observing the association of cognition status and serum NfL level. In our study, more severe cognition decline measured by MMSE was significantly associated with higher serum NfL level. In the acute attack group and remission group, serum NfL level fluctuated with MMSE scale. This confirmed NfL as a neurological dysfunction biomarker clearly from clinical perspective. Likewise, another study over small vessel disease revealed serum NfL correlated with focal neurological symptoms and disability as well^19^. This is not surprising because both the underlying neurological dysfunction and accumulating and progressive cortical injuries resulting from the stroke-like episodes might cause cognition impairment^20^.

Apart from the clinical evaluation, we further explored the correlation of the serum NfL levels with MRI results. The functional MRI is an tool that reflects neurological dysfunction in MELAS, often used in the diagnosis, follow-up and prediction of this disorder^5^. Serum NfL levels were reported to be strongly linear with SLLs volume measured by fMRI in patients with ischemic stroke^21^. Similar results were observed in familial Alzheimer patients^22^. Besides, serum NfL levels were reported to correlated with several metrics of diffusion tensor imaging in patients with diffuse axonal injury and small vessel disease, thus, demonstrating NfL as a potential biomarker for disease progression in neurodegenerative disorders^19, 23, 24^. Specifically, these findings suggested that the serum NfL level may be used in monitoring CNS-associated disorders similar to what multi-functional MRI accomplishes.

Presenting with a progressive and fluctuating disease course, patients with MELAS have often been observed to have dynamically changing lesions upon MRI examinations^25, 26^. Thus, we hypothesized that a change in serum NfL level would be correlated with lesion volume. To this end, we examined two patients with MELAS that were continuously monitored with brain MRI and serum NfL at follow-up that previously exhibited the acute attack and remission phase of the disease. Importantly, the serum NfL concentrations and the lesion volumes in their acute attack were higher than the remission phase comparisons. The serum NfL level fluctuated with the disease status, namely, increasing in the acute attack phase and decreasing in the remission phase. This NfL level was consistent with the lesion volume change based on brain MRI results. Thus, the serum NfL was observed as dynamic biomarker for MELAS in these two patients. In fact, serum NfL was proposed as a dynamic biomarker in other neurological diseases including traumatic brain injury^16^, Huntington's disease^27^ and ischemic stroke^21^. This dynamic characteristic of the serum NfL based on disease status needs to be confirmed in the larger and prospective cohort.

Taken together, these results support the potential utility of serum NfL as a MELAS biomarker that is associated with neurological dysfunction. Importantly, measuring the serum NfL levels provided a new insight into the neurological dysfunction of MELAS. From a clinical perspective, NfL provided the opportunity for prognostic and therapeutic assessment in future studies. The results of this study and the dynamic nature of NfL observed at different MELAS disease states need to be confirmed by larger prospective studies.

## Methods

All methods were in accordance with relevant guidelines and regulations.

### Standard protocol approvals, registrations, and patient consents

This study was approved by the Regional Ethics Committee of Huashan Hospital, Shanghai, China. Written informed consent was obtained from all patients (or guardians of patients) in the study.

### Study participants

This cross-sectional study enrolled patients with MELAS from May 2015 to August 2019. Patients with MELAS were diagnosed by mitochondrial gene testing (using blood sample or muscle tissue) and/or muscle biopsy that met the criteria of the MELAS study committee in Japan^28^. Complete mitochondrial genome sequencing was done in all patients. No neurological diseases or brain injury occurred in HCs. Patients with MELAS were divided into two groups, the acute attack group (MELAS-attack, within 2 weeks after the stroke-like episode) and the remission group (MELAS-remission, 6 months after stroke-like episode without fresh lesion(s) by MRI and new neurological symptoms). The clinical evaluation and blood samples were collected at the same day. Brain multiple functional MRI or conventional MRI were performed within 1 day before or after blood sample was collected. Four blood samples of two patients were collected in the continuous acute attack and remission phase.

### Serum neurofilament light

Serum samples were processed within one hour from collection and stored at − 80 °C. All serum samples evaluated by the Simoa to measure the NfL levels because it is more sensitive than other methods, namely, ELISA and electrochemiluminescence arrays (ECL), as previously described^18^. The NfL calibrators ranged from 0 to 2000 pg/ml. The analytical sensitivity was 0.32 pg/ml. All blood samples had concentrations above these levels. Samples with intra-assay coefficients of variation above 20% were measured multiple times. Sample measurements were conducted in a blinded fashion.

### Cognition evaluation

The Newcastle Mitochondrion Disease Adult Scale (NMDAS) is the most widely used scale for mitochondrion disease. However, the cognition impairment evaluation in this scale was too confused to be completed by Chinese patients. It could not describe the extensiveness of cognition dysfunction. Thus, MMSE was exploited to assess cognition status in patients with MELAS with both acute attack and remission.

### Neuroimaging

Brain multi-model MR scanning was performed within one day before or after blood sample collection and MMSE evaluation. MRI was performed on a 3.0 T scanner (Discovery MR750, General Electric, Boston, MA, USA) equipped with an 8-channel head coil. The high-resolution 3D T1-weighted images were acquired by a brain volume (BRAVO) sequence: TR = 8.2 ms, TE = 3.2 ms, slice thickness = 1.2 mm, flip angle = 12°, slices = 170, FOV = 240 mm × 240 mm, matrix size = 256 × 256. Fluid-attenuated inversion recovery (FLAIR) scans were acquired with the following parameters: TR = 8800 ms, TE = 145 ms, slice thickness = 6 mm, slices = 18, FOV = 240 mm × 240 mm, matrix size = 224 × 224. Diffusion weighted imaging (DWI) scans were also acquired (TR = 3000 ms, TE = 70 ms, slice thickness = 6 mm, slices = 16, FOV = 240 mm × 240 mm, matrix size = 160 × 160).

The SLLs of each patient with MELAS were identified by images from the T1 BRAVO, FLAIR and ASL (arterial spin-labeling), and manually segmented on the T1 BRAVO images slice by slice using the MRIcron software (https://www.nitrc.org/projects/mricron). the SLLs mask images were then spatially normalized to the standard MNI (Montreal Neurological Institute) space using the SPM12 and resampled to 1.5 mm × 1.5 mm × 1.5 mm. Figures were visualized using the BrainNet Viewer (http://www.nitrc.org/projects/bnv/). According to previous studies, acute SLLs showed hyperperfusion and remote lesions showed hypoperfusion on ASL^5, 29^. Therefore, we identified fresh SLLs in patients with the acute attack by ASL and excluded the previous lesions. Then, fresh SLLs in patients with the acute attack and old lesions in remission were segmented on the T1 BRAVO images referencing by ASL, respectively. The volumes of the acute SLLs and old lesions were calculated using custom-written scripts using the MATLAB software.

### Statistics analysis

Normality of the data was verified. Group comparisons were performed. The post hoc test was used to analyze the differences of serum NfL level among patients with MELAS in the acute attack and remission phase as well as HCs. The correlation of NfL concentration and MMSE score was tested by the Pearson correlation test. The correlation of NfL concentration and SLLs volume was analyzed by the Spearman rank correlation test. Normal distribution data was processed and the mean ± SD was calculated. Non-normal distribution data was processed and the median and interquartile range (IQR) were assessed. All tests were two-sided. Statistical significance was determined at p < 0.05. Statistics and charts were produced in SPSS 24.0 and the GraphPad Prism 8.0.2 software.

## Data Availability

Data from this study are available upon request.
