# Author Correction: Neurofilament light is a novel biomarker for mitochondrial encephalomyopathy, lactic acidosis, and stroke-like episodes

**DOI:** 10.1038/s41598-021-94591-w

**Published:** 2021-07-28

**Authors:** Yong‑Sheng Zheng, Chong Sun, Rong Wang, Ne Chen, Su‑Shan Luo, Jian‑Ying Xi, Jia‑Hong Lu, Chong‑Bo Zhao, Yu‑Xin Li, Lei Zhou, Jie Lin

**Affiliations:** 1 Department of Neurology, Huashan Hospital, Fudan University, 12 Middle Urumqi Road, Shanghai, 200040 China; 2 Department of Radiology, Huashan Hospital, Fudan University, 12 Middle Urumqi Road, Shanghai, 200040 China

Correction to: *Scientific Reports* https://doi.org/10.1038/s41598-021-81721-7, published online 21 January 2021

In the original version of this Article Yong-Sheng Zheng, Chong Sun and Rong Wang were omitted as equally contributing authors.

The original Article has been corrected.

